# Primary Hyperparathyroidism and Pancreatitis: A Rare Association with Multiple Facets

**DOI:** 10.1155/2016/7294274

**Published:** 2016-09-27

**Authors:** I. Diallo, C. A. Fall, B. Ndiaye, M. Mbaye, I. Diedhiou, A. R. Ndiaye, P. S. Diawara, F. Fall, P. S. Mbaye, S. B. Gning

**Affiliations:** ^1^Department of Internal Medicine and Hepato-Gastroenterology, Hôpital Principal de Dakar, Dakar, Senegal; ^2^Department of Medical Imaging, Hôpital Principal de Dakar, Dakar, Senegal; ^3^Department of Biology, Hôpital Principal de Dakar, Dakar, Senegal

## Abstract

Primary hyperparathyroidism (PHPT) is rarely associated with the occurrence of acute or chronic pancreatitis. Hypercalcemia plays a major role in the pathogenesis. We report five cases of pancreatitis revealing PHPT.* Patients and Methods*. This is a retrospective study of 4 years, including all patients admitted to intensive care unit or gastroenterology department, for an acute or chronic pancreatitis revealing primary hyperparathyroidism.* Results*. We included 5 patients, all female, with mean age 54 years [40–76 years]. The PHPT was in all cases revealed by acute pancreatitis (AP). This one was oedematous in four cases and severe in one case. It occurred twice in calcified chronic pancreatitis (CCP). There was hypercalcemia in all cases. The PHPT was associated with a high rate of parathyroid hormone in 4 cases. The secreting lesion was an adenoma in 5 cases. Two patients had in addition bilateral renal calcifications. The outcome was favorable in 4 patients among whom 3 have had parathyroid surgery. A death was noted by superinfection of necrosis in the case of severe AP.* Conclusion*. The occurrence of pancreatitis during hyperparathyroidism is rare. Normal or elevated calcemia during acute or chronic pancreatitis should always get attention.

## 1. Introduction

The causes of pancreatitis are largely dominated by gallstones and alcohol. Primary hyperparathyroidism (PHPT) is rarely associated with the development of pancreatitis. It is considered as well a cause of acute or chronic pancreatitis (AP or CP). Hypercalcemia secondary to the secretion of parathyroid hormone (PTH) plays a major role in the pathogenesis, but other mechanisms may be involved. The manifestations are polymorphic. We report five cases of pancreatitis revealing PHPT.

## 2. Patients and Methods

This is a retrospective and descriptive study from January 2011 to December 2014, including all patients admitted to intensive care units or gastroenterology department at Hôpital Principal de Dakar (HPD) for an acute or chronic pancreatitis revealing a PHPT.

The diagnosis of pancreatitis was retained in the presence of at least 2 of the 3 following elements: abdominal pain, levels of serum amylase, or lipase greater than 3 times the normal, or characteristic aspects of AP (oedema or pancreatic necrosis, acute necrosis collection) or CP (pancreatic atrophy or hypertrophy, pancreatic calcifications, ductal abnormalities, and cysts) at imaging. For PHPT, hypercalcemia associated with increased PTH levels posed diagnosis. A search of the secretory lesion was made by imaging.

Other causes of acute or chronic pancreatitis were sought, including cholelithiasis, alcohol consumption, or hypertriglyceridemia. Patients with another etiology that could explain pancreatitis were not retained. The epidemiological, clinical, and paraclinical outcomes of the patients were noted.

## 3. Results

During the study period, 61 patients were hospitalized for pancreatitis (54 AP and 7 CP). Among them, 5 patients had a pancreatitis revealing PHPT (8%). Hospital prevalence of this association was 1.25 cases per year. All patients were female, with mean age 54 years [40–76 years]. One patient was diabetic and hypertensive, with stroke sequelae like left hemiparesis, another was diabetic, and a third was hypertensive. No patient had consumed alcohol and one was smoking 40 pack-years.

Revealing clinical symptoms were abdominal pain in all cases associated with vomiting in two patients. One patient was overweight with a body mass index (BMI) at 27.34 kg/m^2^ and two were obese with BMI, respectively, at 32 and 34 kg/m^2^. Clinical examination found in all cases a sensitive but flexible abdomen. One patient had hypovolemic shock.

AP was suspected and confirmed by dosage of pancreatic enzymes and imaging. Lipasemia was high in four cases with a rate of more than 40 times normal in the case of AP that had occurred on a normal pancreas. Amylasemia was also high in both cases where it was performed. Abdominal CT scan showed a homogeneous pancreatic hypertrophy without necrosis (*n* = 2) and a heterogeneous pancreatic hypertrophy associated with flows of necrosis and a peritoneal effusion (*n* = 1). There were pancreatic calcifications and small pseudocysts in two cases associated with infiltration of the peripancreatic fat (*n* = 1) or with atrophy of the pancreas (*n* = 1). In total, the AP was benign in four cases and severe in one patient. It occurred twice in CP.

PHPT was in all cases revealed by an AP. Searching for an hyperparathyroidism was achieved after eliminating the most common causes of AP. No patient was taking alcohol. Cholelithiasis or early change in liver function tests was not found enabling us to eliminate a biliary etiology. Serum calcium was high in all cases with an average of 119.6 mg/L (104–130). There was no context of neoplasia and serum protein electrophoresis showed no monoclonal peak or hypogammaglobulinemia. The diagnosis of PHPT was confirmed by the dosage of PTH which was high in 4 cases with a mean value of 331 ng/L (199–536) and normal in a patient. Patients characteristics are summarized in Table 1.

**Figure 1:**
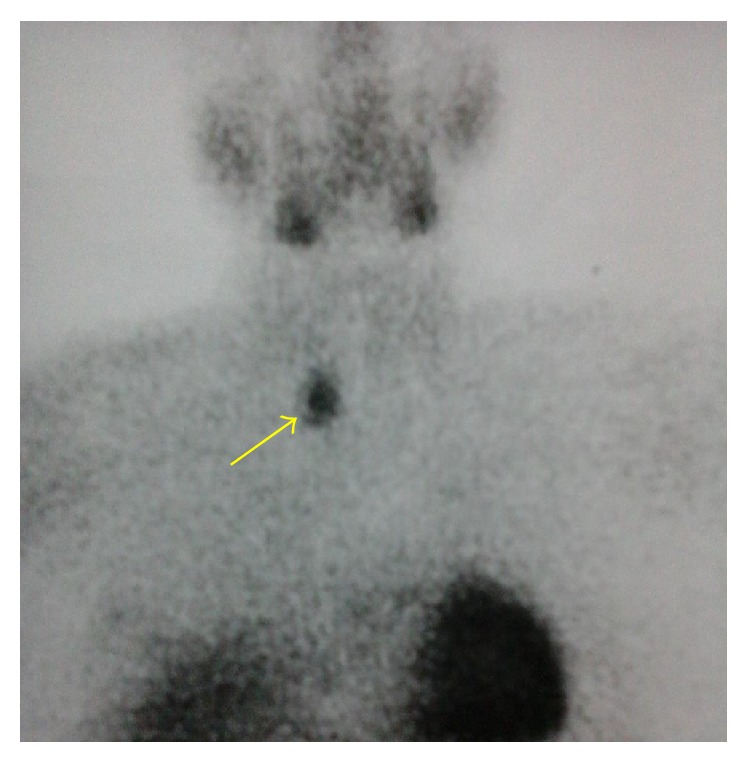
Hyperfunctional parathyroid adenoma in retrotracheal and right paraesophageal position (arrow).

The secreting lesion was found in all cases by ultrasound or computed tomography. This was a parathyroid nodular lesion in 3 patients. In one case, the nodule was lateroesophageal right at the cervicothoracic hole whose character of hyperfunctional parathyroid was confirmed by MIBI-Tc-99m scintigraphy (Figure 1). For the last patient, a parathyroid lesion or suspicious cervical or thoracic lesion has not been found at scanner and MRI. But the abdominal CT scan showed a left adrenal lesion whose appearance might suggest an ectopic parathyroid lesion. However, she could not get a scintigraphy for confirmation. Thus, the etiology of hyperparathyroidism was adenoma with variable size in our five patients with parathyroid localization in 3 cases and ectopic in 2 cases. Multiple endocrine neoplasia (MEN) was investigated in all patients with the dosage of calcitonin and urinary methoxyamines that were negative in all cases.

Furthermore, two patients who had an AP on CP had bilateral renal lithiasis. These lesions were responsible for a deterioration of renal function with a renal clearance, respectively, to 19 and 20 mL/min.

Therapeutically, all patients initially received symptomatic management of the AP. Only one patient received bisphosphonates awaiting surgery. An explorative cervicotomy was performed in three patients with resection of the parathyroid adenoma. The patient who is 74 years old did not have surgery due to significant comorbidities. The death was noted in one who had severe AP, following a superinfection of necrosis flows.

The outcome was favorable in 3 patients operated on, with normalization of PTH and serum calcium and no recurrence of the AP. The other one with CP and PHPT, who was not operated on, presented recurrent minor abdominal pain relieved by simple painkillers.

## 4. Discussion

The occurrence of pancreatitis secondary to PHPT is not so rare, with a prevalence of 3.6% (1.5 to 15.3%) [1], as shown by the rate of 8% of this association found in our study. Our hospital prevalence of 1.25 cases per year illustrates this rarity. The discovery of a pancreatic disease increases by 33 the risk of having a PHPT [2], while the existence of PHPT would multiply by a factor of 10 to 30 the risk of pancreatitis [1–3]. Some authors, however, refute this increased risk of pancreatic disease with PHPT. Indeed, they found in the general population, a prevalence of pancreatitis lower or equivalent in patients with PHPT than in the control group [4, 5].

Pancreatitis occurs at an advanced stage of parathyroid disease [1], which would explain the low prevalence of this association in the developed countries, PHPT being diagnosed earlier. The mean age at diagnosis is variable, but patients are older than those with only PHPT [2, 6]. They are young adults in midlife in the cases described in India and Latin America [2, 7, 8], while patients were older (60–70 years) in the United States and France [5, 6]. The male sex is predominant in most studies with about 60 and 70% of men [2, 7, 8], in contrast to patients with only a single PHPT. Our series has the feature of having only women with an average age of 54 years.

The occurrence mechanism of pancreatitis during PHPT remains controversial but may be related to hypercalcemia, only statistically significant factor associated [6]. Shah suggested that a high calcium level in more than 1.3 times normal was associated with a risk of occurrence of an AP [9]. Serum calcium is generally higher in patients with pancreatitis during PHPT than those who have only PHPT [2, 5, 10]. For CP complicating PHPT, serum calcium is also higher than in the CP of alcoholic or idiopathic origin [11]. In the CP, this mechanism seems obvious ahead of calcium deposits in the absence of other causes.

Hypercalcemia would act by several mechanisms: increased level of calcium in pancreatic juice at the origin of activation of trypsinogen to trypsin; activation of pancreatic enzymes through the lysosomal system and hydrolases; calcium precipitation and formation of protein plugs responsible for upstream pancreatitis. The direct toxic action of PTH on the pancreas is mentioned, but pancreatitis is not usually found in dialysis patients with elevated PTH [6], and PTH is not higher in patients with pancreatitis in the course of PHPT [2, 6]. A genetic risk factor has also been found. Indeed, mutation of SPINK1 gene (Serine Protease Inhibitor Kazal type I) and CFTR gene (Cystic Fibrosis Transmembrane Conductance Regulator) was found more often in patients with PHPT who developed an AP [10]. In our patients, hypercalcemia present in all cases despite pancreatitis seems to be the main mechanism.

Thus, viewing these different mechanisms, the association of PHPT and pancreatitis can take many forms. So, Jacob et al. proposed a classification of this association which can be in 4 forms [2]: PHPT revealed by AP, PHPT revealed by recurrent AP without CP, PHPT revealed by a CP with or without pancreatic calcifications, or PHPT complicated by AP in the postoperative period.

The circumstances of discovery are dominated by acute pancreatitis which is revealing in 75% of cases [1]. The diagnosis of AP is classic in front of abdominal pain, with the dosage of pancreatic enzymes and imaging. There may be varying severity of AP. In our patients AP was benign in 4 of 5 cases, unlike the series of Gupta et al. where 4 of the 5 patients had necrotic pancreatitis [7]. This AP may occur on normal pancreas or CP. In the meta-analysis of Bai et al., there were 35.3% of CP [1], and in our study we found 40% of CP.

The PHPT was diagnosed in all of our patients in the course of etiological research of this pancreatitis and hypercalcemia. The diagnosis is made by the PTH dosage which was elevated in 4 of our patients. PTH in patients with pancreatitis on PHPT is not higher than among those with only PHPT [2, 5, 6]. The localization of the secretory lesion was made by the anterior cervical ultrasound and CT scan. One patient underwent MIBI-Tc-99m scintigraphy, in front of a suspicious cervical lesion and normal PTH levels. This examination confirmed the hyperfunctional character of this parathyroid laterocervical nodule. Parathyroid lesion was in any case an adenoma in our series, as in Indian series [2, 7, 12]. It was ectopic in two cases out of 5. Parathyroid adenoma is found in 58–79% of cases in Western literature [6, 8] and hyperplasia is found in 12–21% of cases [6, 8], while parathyroid carcinoma is exceptional in these cases.

The discovery of PHPT imposes the research of other events related to hypercalcemia. Due to the advanced parathyroid disease, these lesions are more often found in patients with pancreatitis on PHPT than those having only PHPT. Thus, nephrolithiasis is present in 42–46% of cases [2, 8], nephrocalcinosis is present in 30% [2], and gallstones are present in 35% of cases [8]. Badhada showed in his study that nephrocalcinosis, gallbladder or kidney stones, crises of renal colic, bone disease, and psychiatric disorders were more frequent during a CP due to PHPT than in CP of alcoholic or idiopathic origin [11]. Our two patients which had CP had also urolithiasis with impaired renal function indicating a chronicity of the symptomatology. Multiple endocrine neoplasia, not found in our patients, should also be sought, because it is present in some cases [6, 8].

Treatment in patients with pancreatitis on PHPT first rests on a management of the AP which can be fatal. Indeed, one of our patients who had a severe AP died before benefiting from parathyroidectomy. Pending parathyroid surgery, the management of serum calcium may be necessary based on rehydration and bisphosphonates as in our third patient.

Surgical resection of the secreting lesion causes a collapse of postoperative PTH levels [3] and a decrease of serum calcium which must be verified intraoperatively. This treatment prevents recurrence of AP in all cases [2, 8, 11]. However, it causes the disappearance of pancreatic pains in case of CP [11], but without resolution of chronic pancreatitis [2, 13]. In our patients, surgery was performed in three cases with normalization of PTH and serum calcium and no recurrence of the AP. The one that was not operated on because of comorbidities presented moderate abdominal pain relieved by analgesics type 1.

## 5. Conclusion

The occurrence of pancreatitis during a hyperparathyroidism is rare. Normal or higher calcemia during acute or chronic pancreatitis should always draw attention and be subject to complementary explorations in search of endocrine or malignant cause.

## Figures and Tables

**Table 1:** Patients, pancreatitis, and PHPT characteristics.

*N*	Sex	Age	Amylase	Lipase	Type of pancreatitis	Calcemia	PTH	Secretory parathyroid lesion
1	F	40	640 (7, 8 N)		Benign AP on CP	130	536 (8, 2 N)	Right parathyroid adenoma of 25 × 23 mm
2	F	43		3694 (61 N)	Severe AP	119	199 (3 N)	Left parathyroid adenoma of 1.6 cm × 1.4 cm
3	F	57		2466 (41 N)	Benign AP	128	61	Right lateroesophageal parathyroid adenoma at the cervicothoracic hole of 27 × 12 mm
4	F	76		419 (7 N)	Benign AP on CP	104	231 (4, 8 N)	Left ectopic (adrenal?) parathyroid adenoma of 31 × 35 × 63 mm
5	F	55	1114 (13, 6 N)	279 (4, 7 N)	Benign AP	117	360 (5, 5 N)	Right parathyroid adenoma of 7 mm
